# Supplementary figures and images for: Nanosecond pulsed electric fields induce the integrated stress response via reactive oxygen species-mediated heme-regulated inhibitor (HRI) activation
Source: PLoS One. 2020 Mar 10;15(3):e0229948. doi: 10.1371/journal.pone.0229948 (PMC7064201; doi:10.1371/journal.pone.0229948)

A

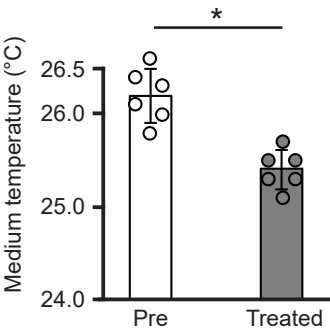

B

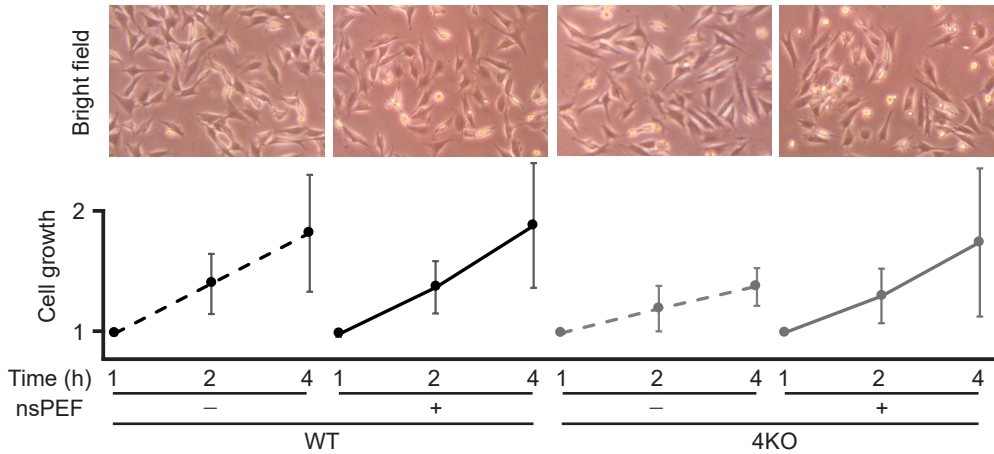

Supplement: S1 Fig — (A) Measurements of medium temperature before and after nsPEFs treatment. Error bars show the means ± SEM (n = 6, *P < 0.05). (B) Representative images and quantification of viability in WT or 4KO cells at 4 h with mock or nsPEF treatment. Cell viability was detected using WST-8 reagent, and the values are shown as the mean as the mean ± SEM. (PDF) [file pone.0229948.s001.pdf]

A

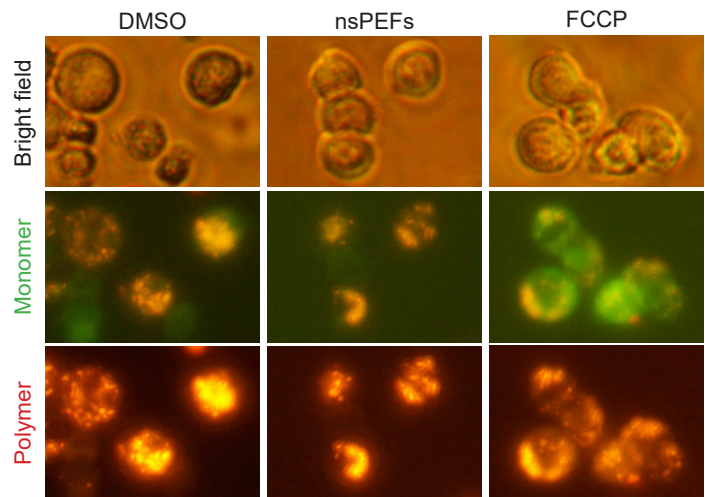

B

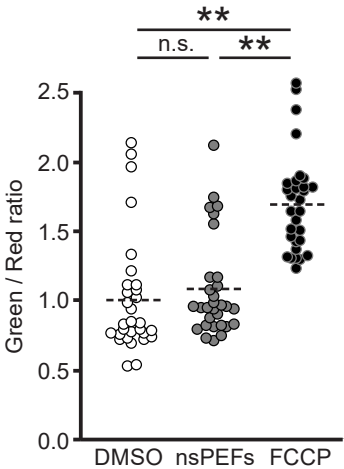

C

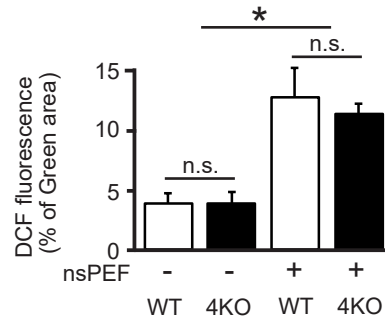

D

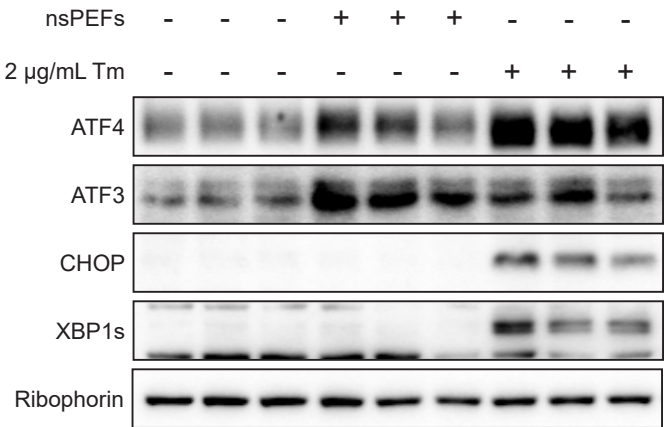

E

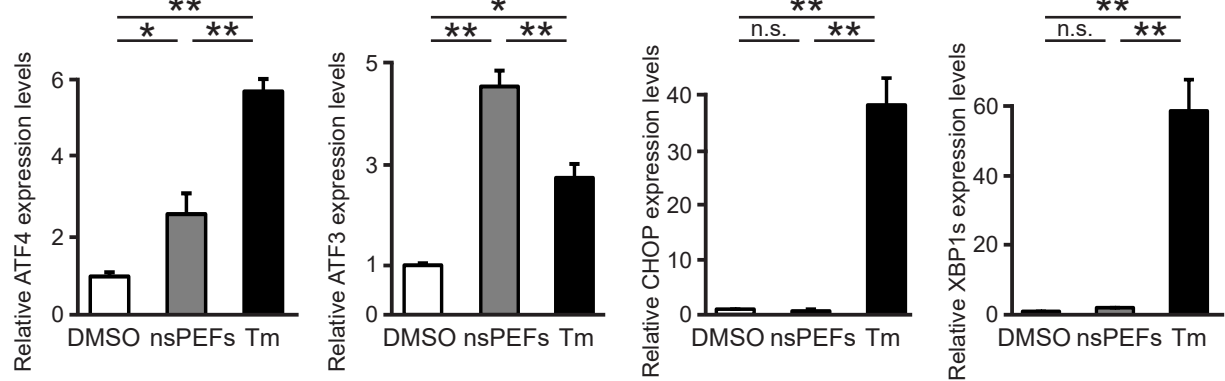

Supplement: S2 Fig — (A) Representative images of JC-1 dye stained cells treated with nsPEF treatment (40 shots of 70-ns duration and 30-kV/cm electric fields) or a mitochondrial uncoupler FCCP used as positive control. Green fluorescence represents the monomeric form of JC-1, indicating dissipation of mitochondrial membrane potential. (B) Quantification of Green (JC-1 monomer)/Red (JC-1 polymer) fluorescence ratio in cells 1 h after nsPEF treatment (40 shots of 70-ns duration and 30-kV/cm electric fields) (n = 30, **P < 0.01, n.s. = not significant). (C) Quantification of intracellular ROS levels in nsPEF-treated WT or 4KO MEFs using the CM-H2DCFDA fluoroprobe. Error bars show the means ± SEM (n = 3–5, *P < 0.05, n.s. = not significant). (D) Representative immunoblots of ATF4, ATF3, CHOP, XBP1s, and Ribophorin 1 h after treatment with the nsPEFs and 2 μg/mL Tm in WT Hap1 cells. (E) Densitometry quantification of ATF4, ATF3, CHOP, and XBP1s expression were normalized to the Ribophorin expression as the mean + SEM (n = 3, *P < 0.05, **P < 0.01, n.s. = not significant). (PDF) [file pone.0229948.s002.pdf]
