# Supplementary material for: Nanosecond pulsed electric fields induce the integrated stress response via reactive oxygen species-mediated heme-regulated inhibitor (HRI) activation
Source: PLoS One. 2020 Mar 10;15(3):e0229948. doi: 10.1371/journal.pone.0229948 (PMC7064201; doi:10.1371/journal.pone.0229948)
Supplement: S1 Data — (PPTX) [file pone.0229948.s003.pptx]

## Slide 1
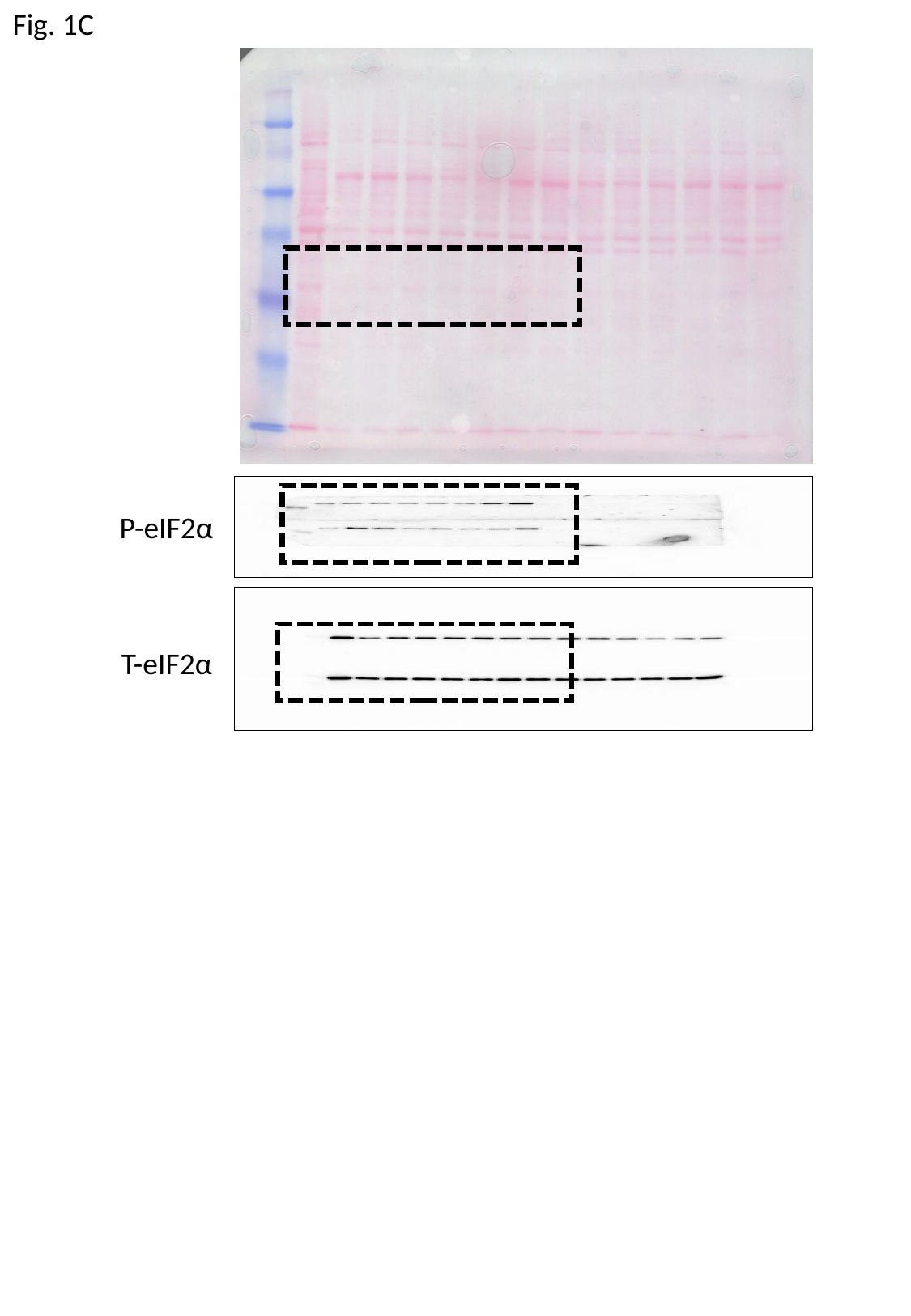

Fig. 1C
P-eIF2α
T-eIF2α

## Slide 2
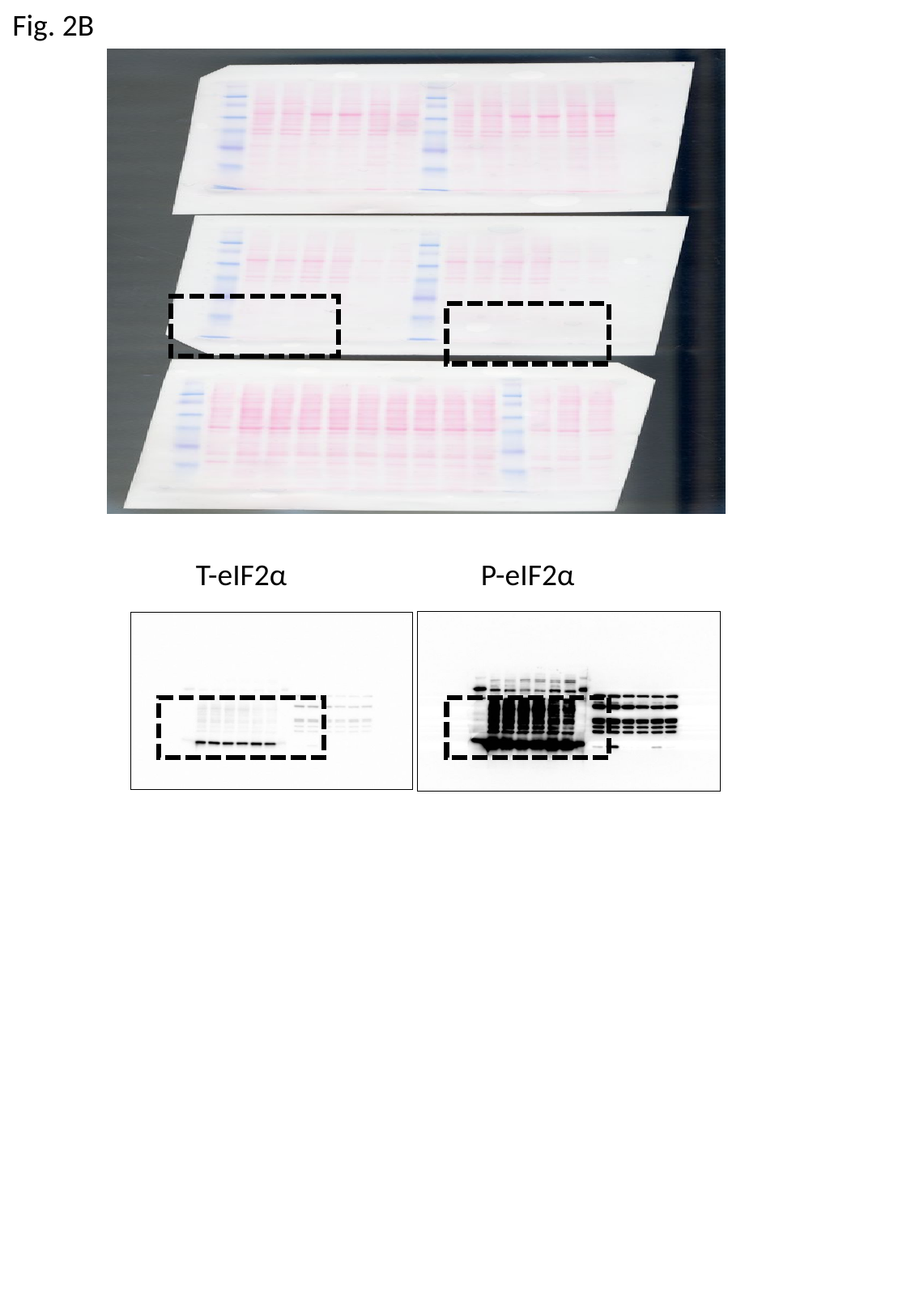

Fig. 2B
T-eIF2α
P-eIF2α

## Slide 3
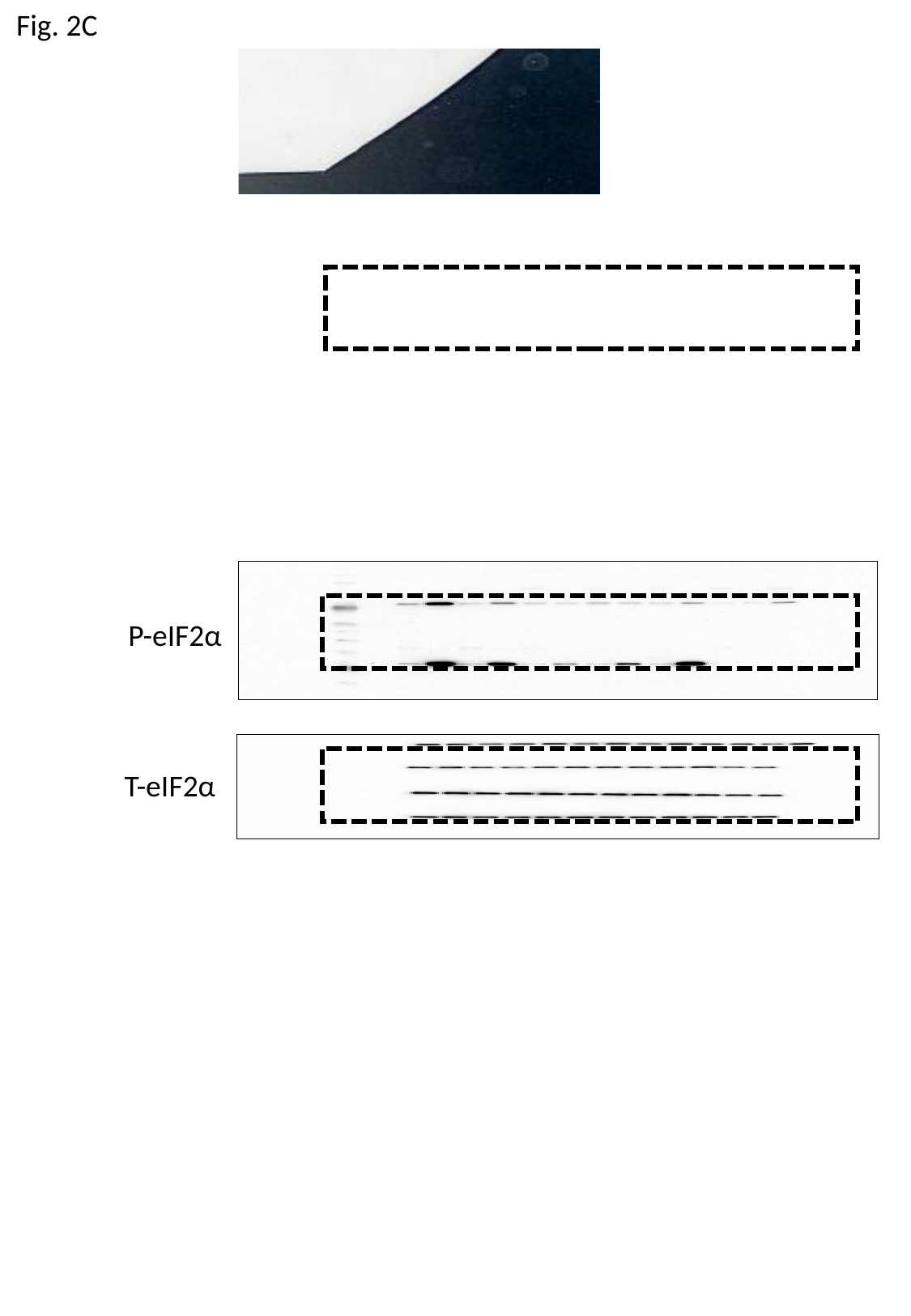

Fig. 2C
P-eIF2α
T-eIF2α

## Slide 4
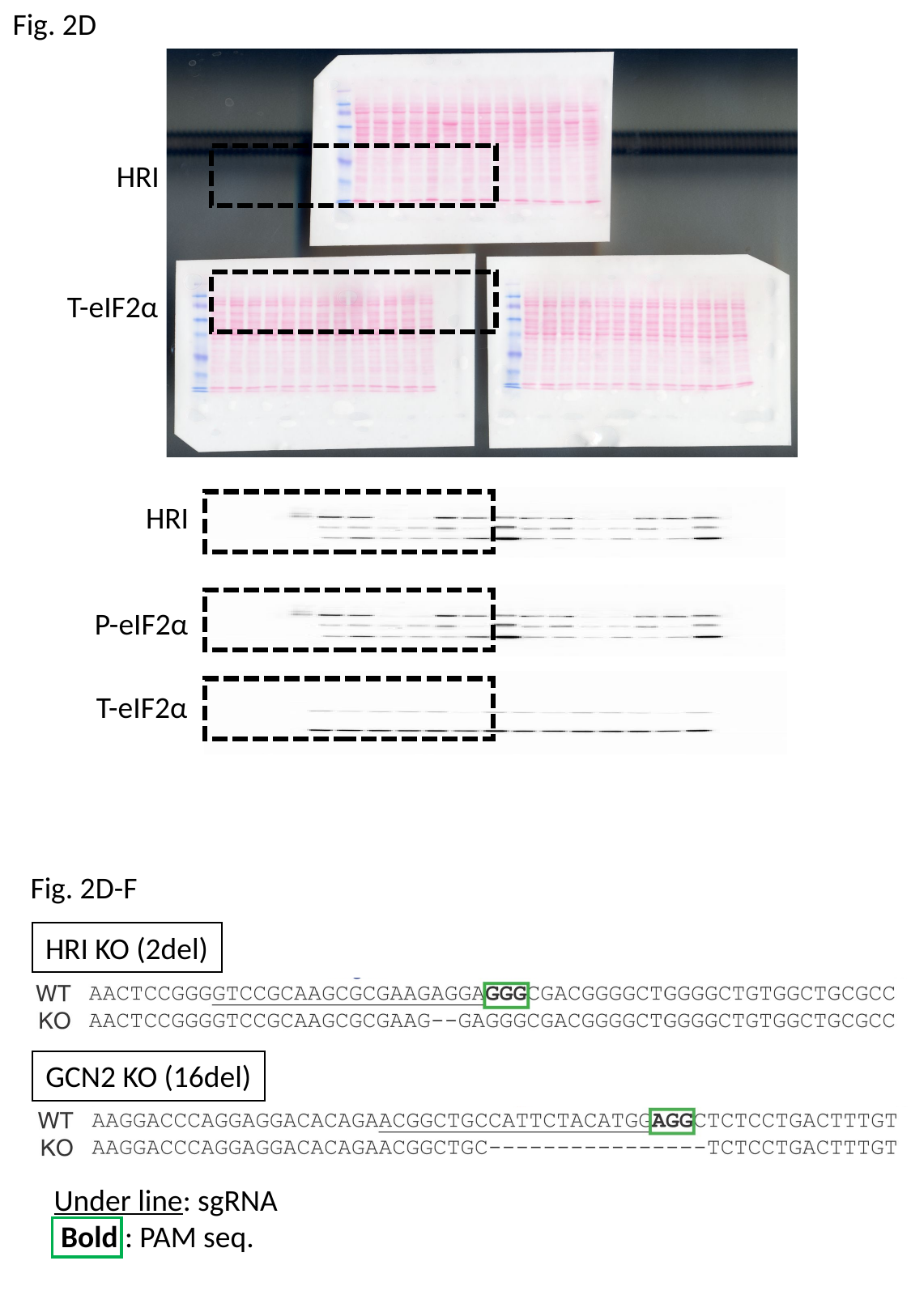

Fig. 2D
HRI
T-eIF2α
HRI
P-eIF2α
T-eIF2α
Fig. 2D-F
HRI KO (2del)
GCN2 KO (16del)
Under line: sgRNA
 Bold : PAM seq.

## Slide 5
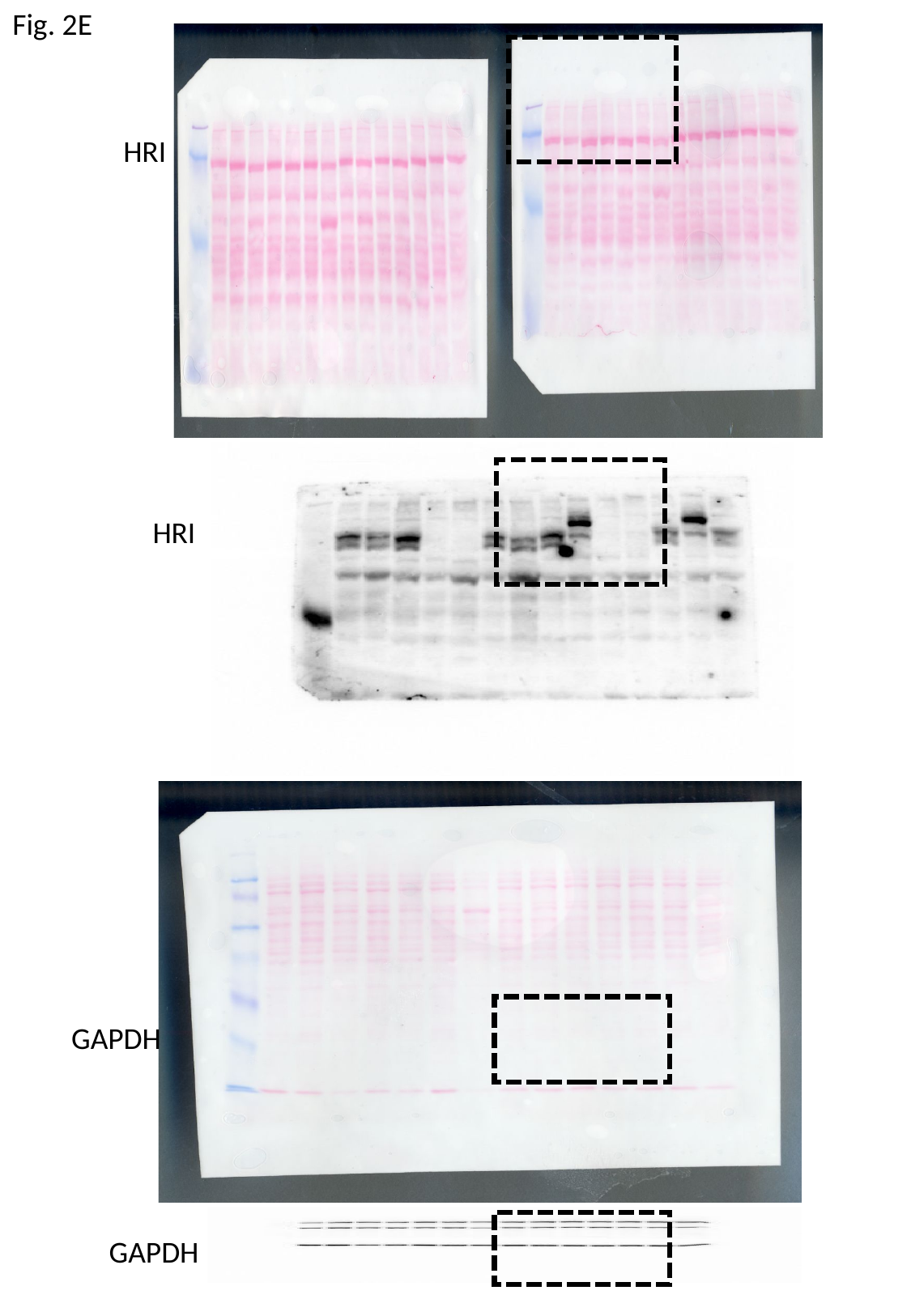

Fig. 2E
HRI
HRI
GAPDH
GAPDH

## Slide 6
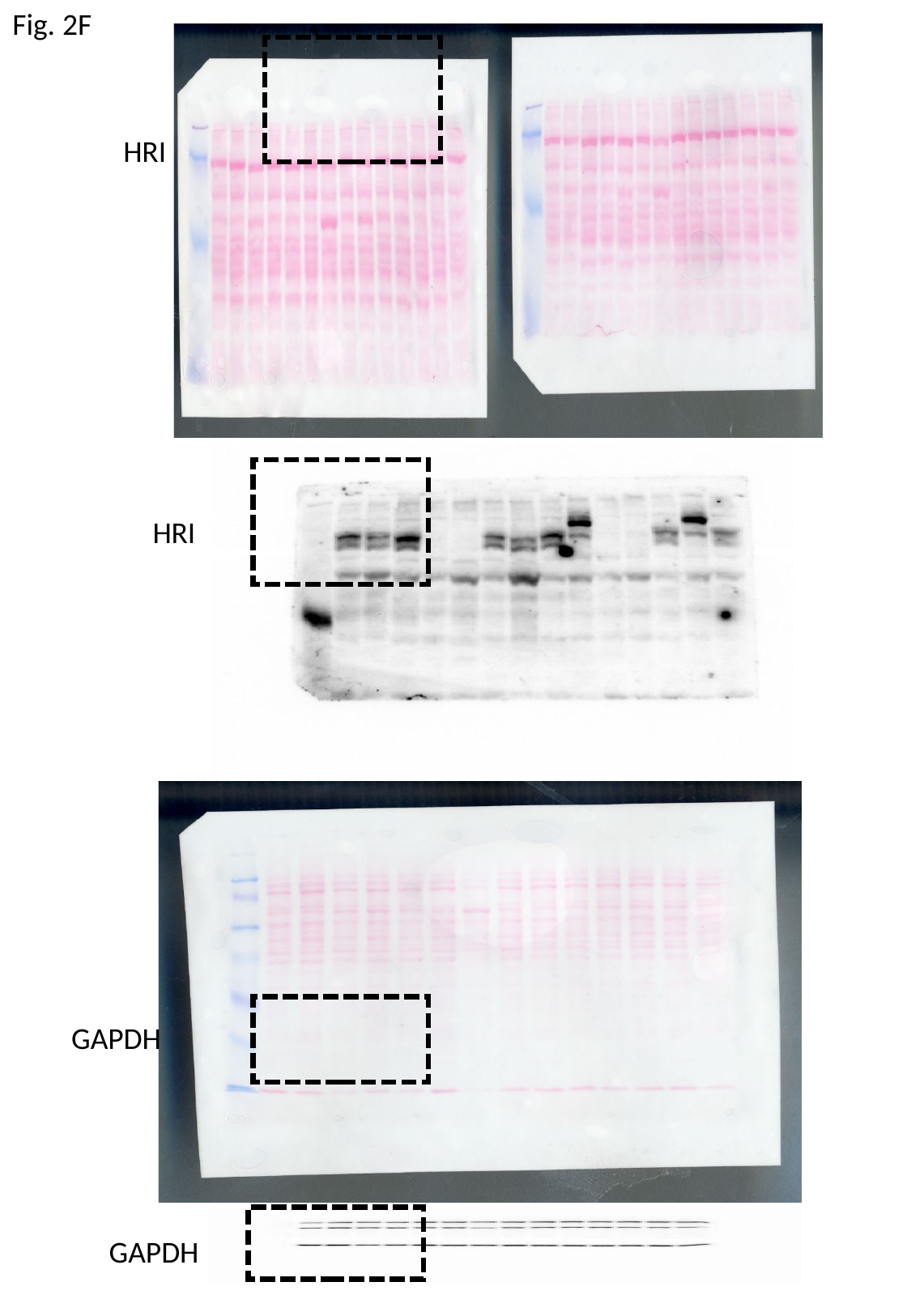

Fig. 2F
HRI
HRI
GAPDH
GAPDH

## Slide 7
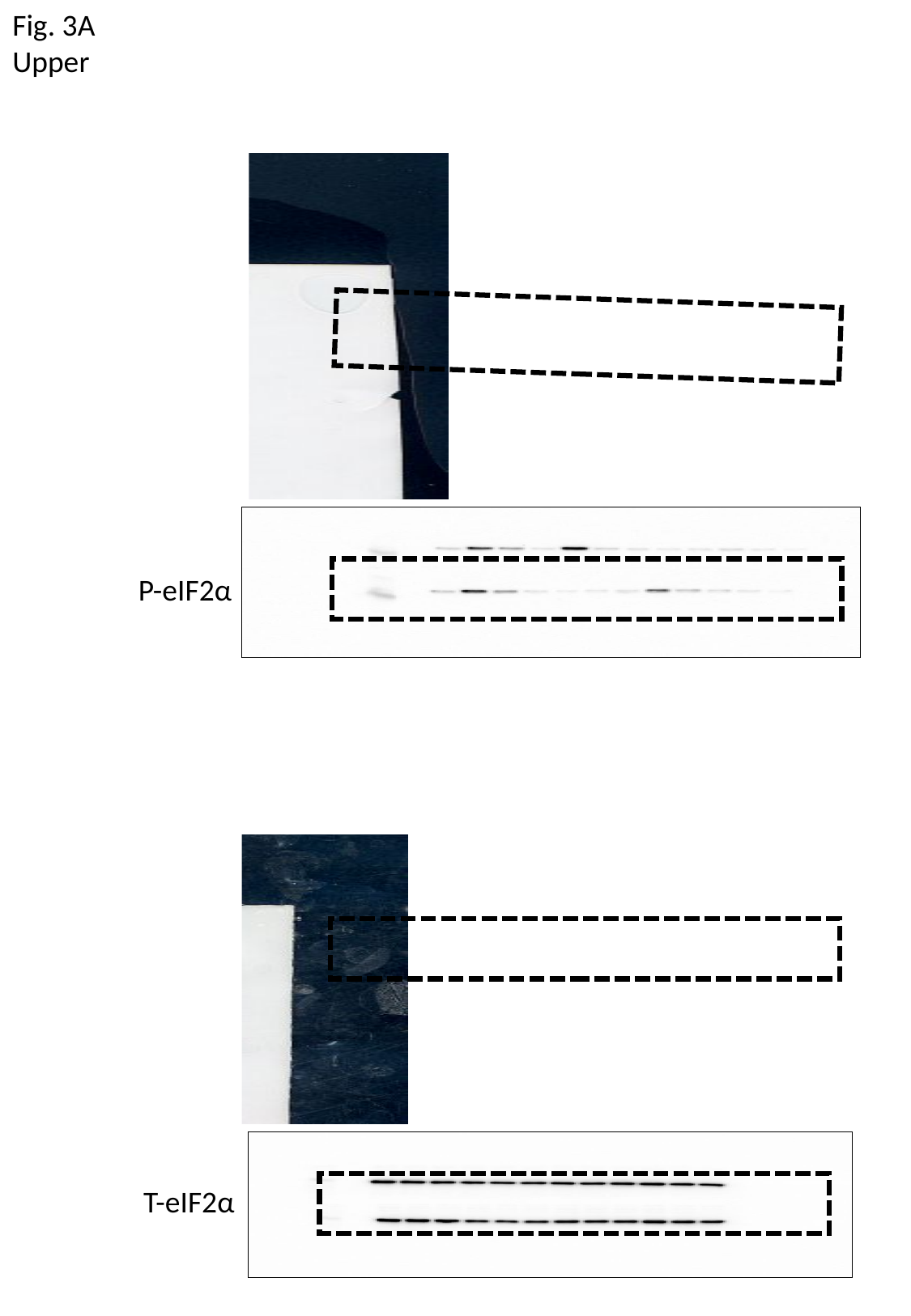

Fig. 3A
Upper
P-eIF2α
T-eIF2α

## Slide 8
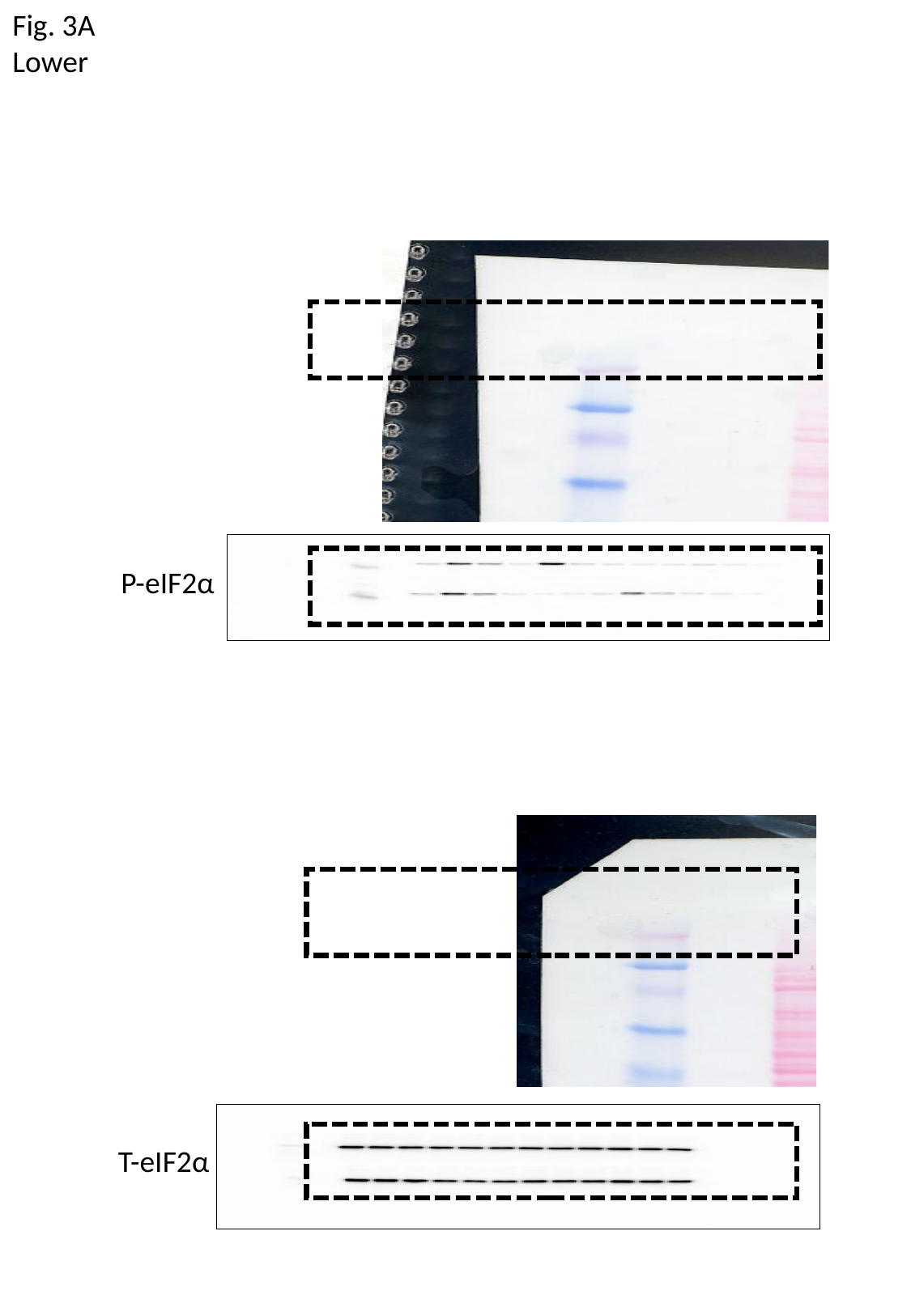

Fig. 3A
Lower
P-eIF2α
T-eIF2α

## Slide 9
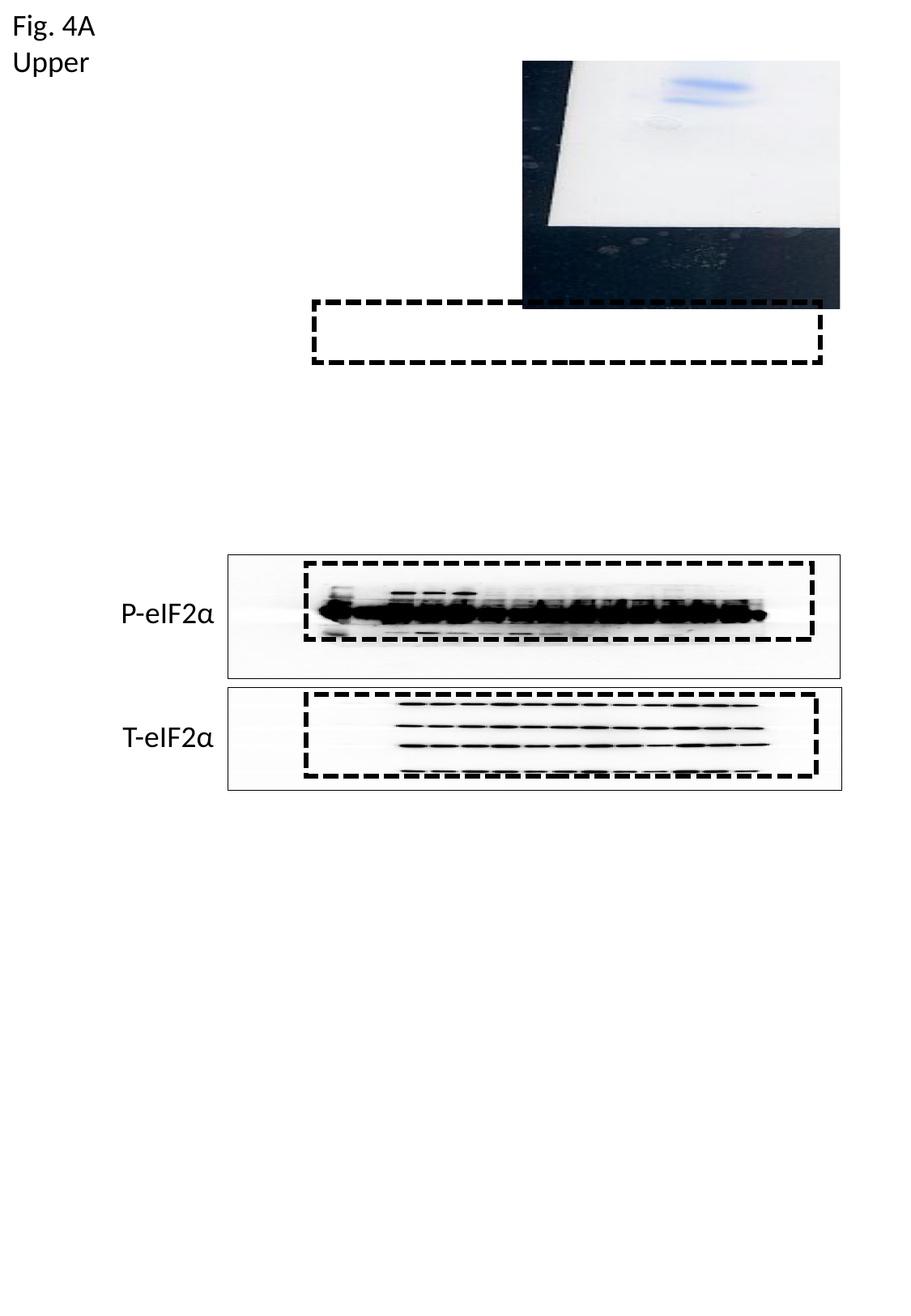

Fig. 4A
Upper
P-eIF2α
T-eIF2α

## Slide 10
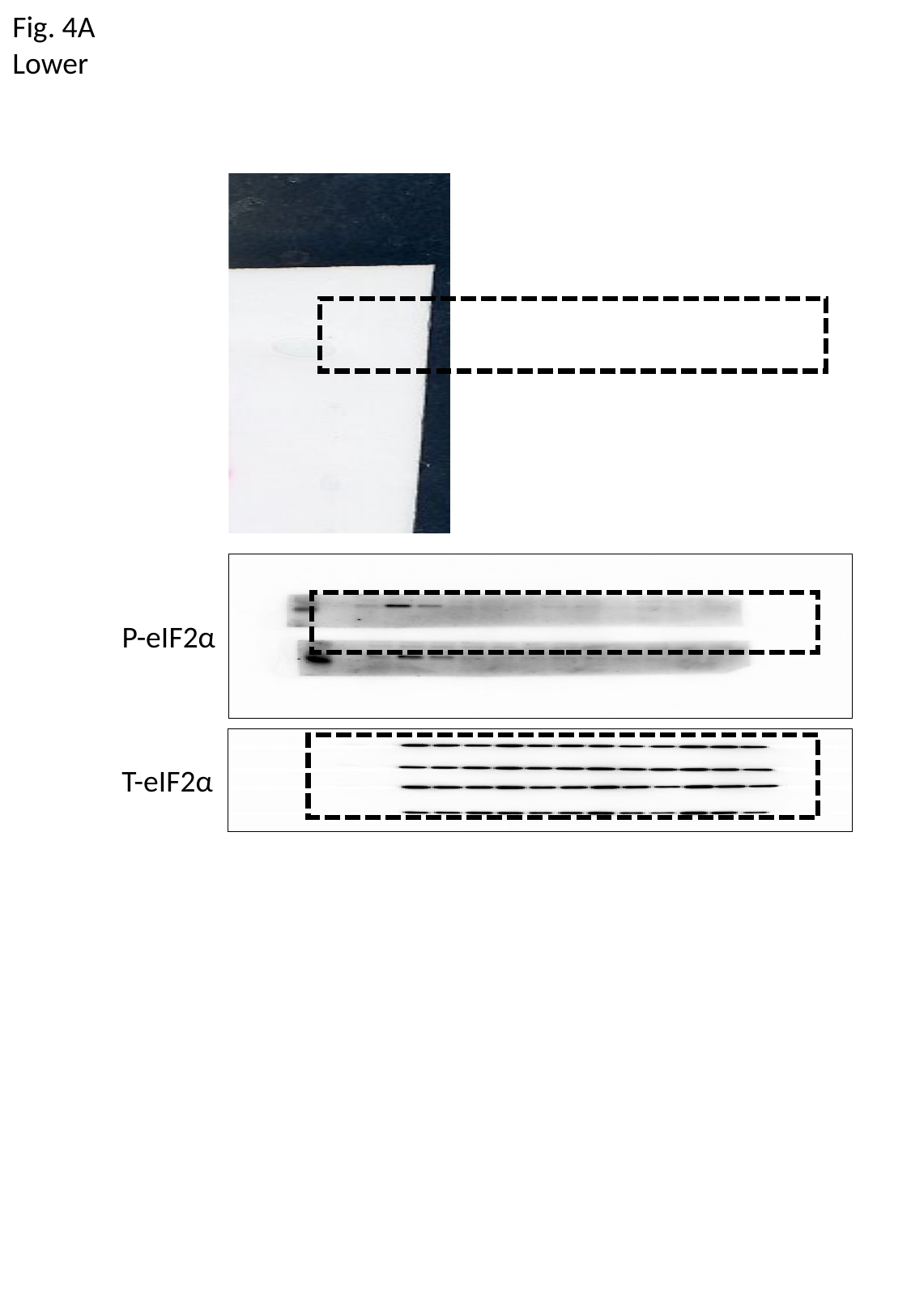

Fig. 4A
Lower
P-eIF2α
T-eIF2α

## Slide 11
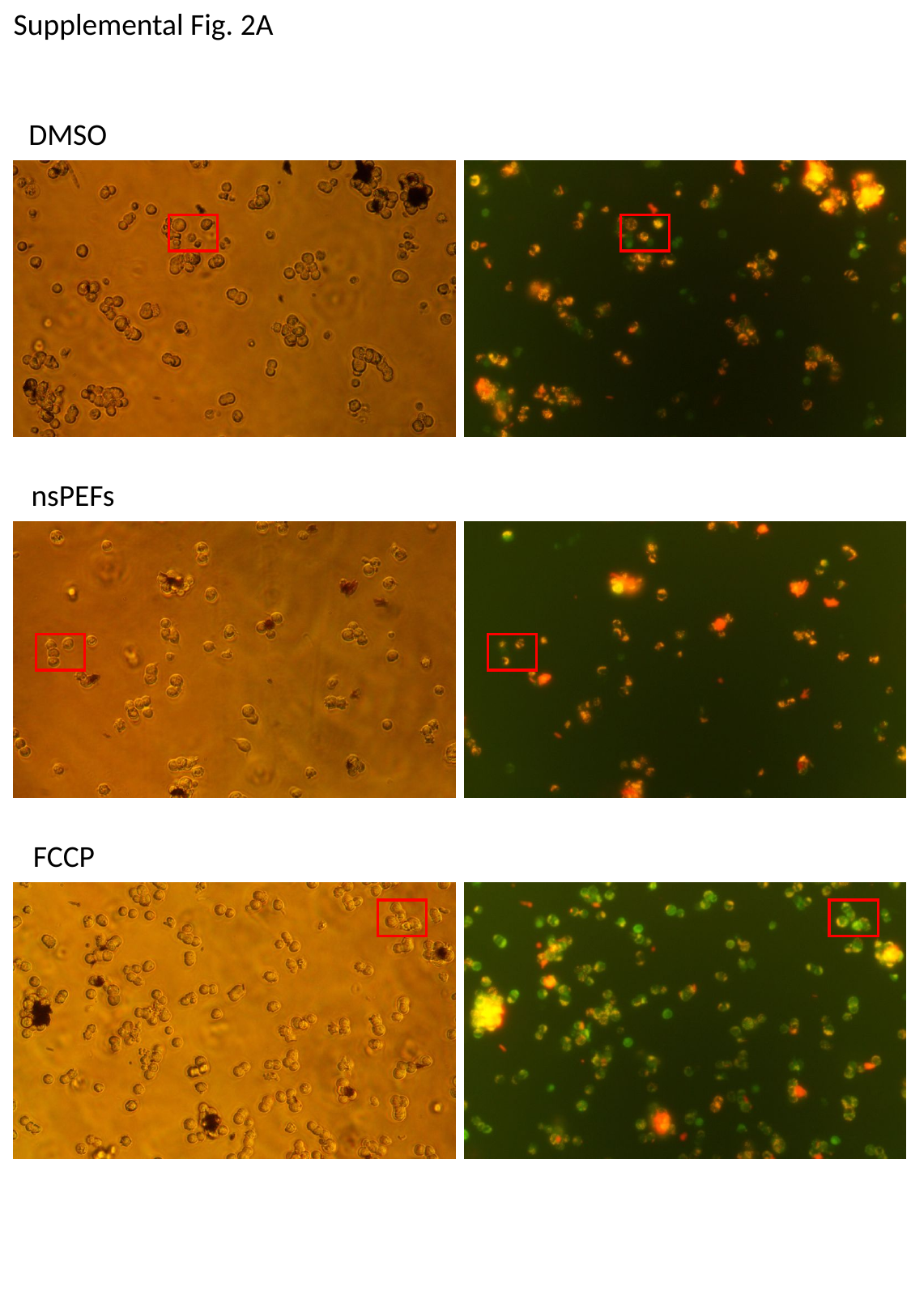

Supplemental Fig. 2A
DMSO
nsPEFs
FCCP

## Slide 12
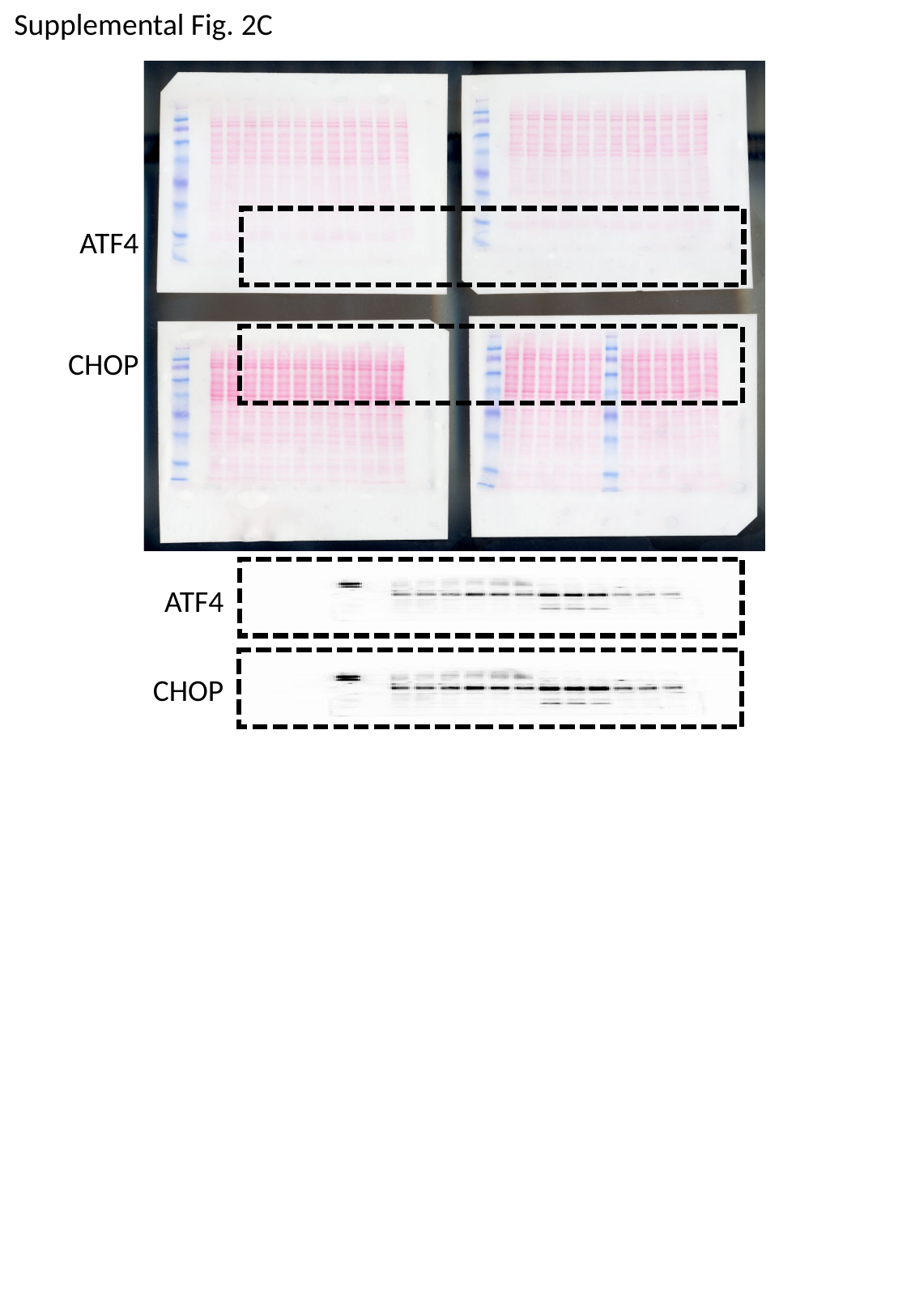

Supplemental Fig. 2C
ATF4
CHOP
ATF4
CHOP

## Slide 13
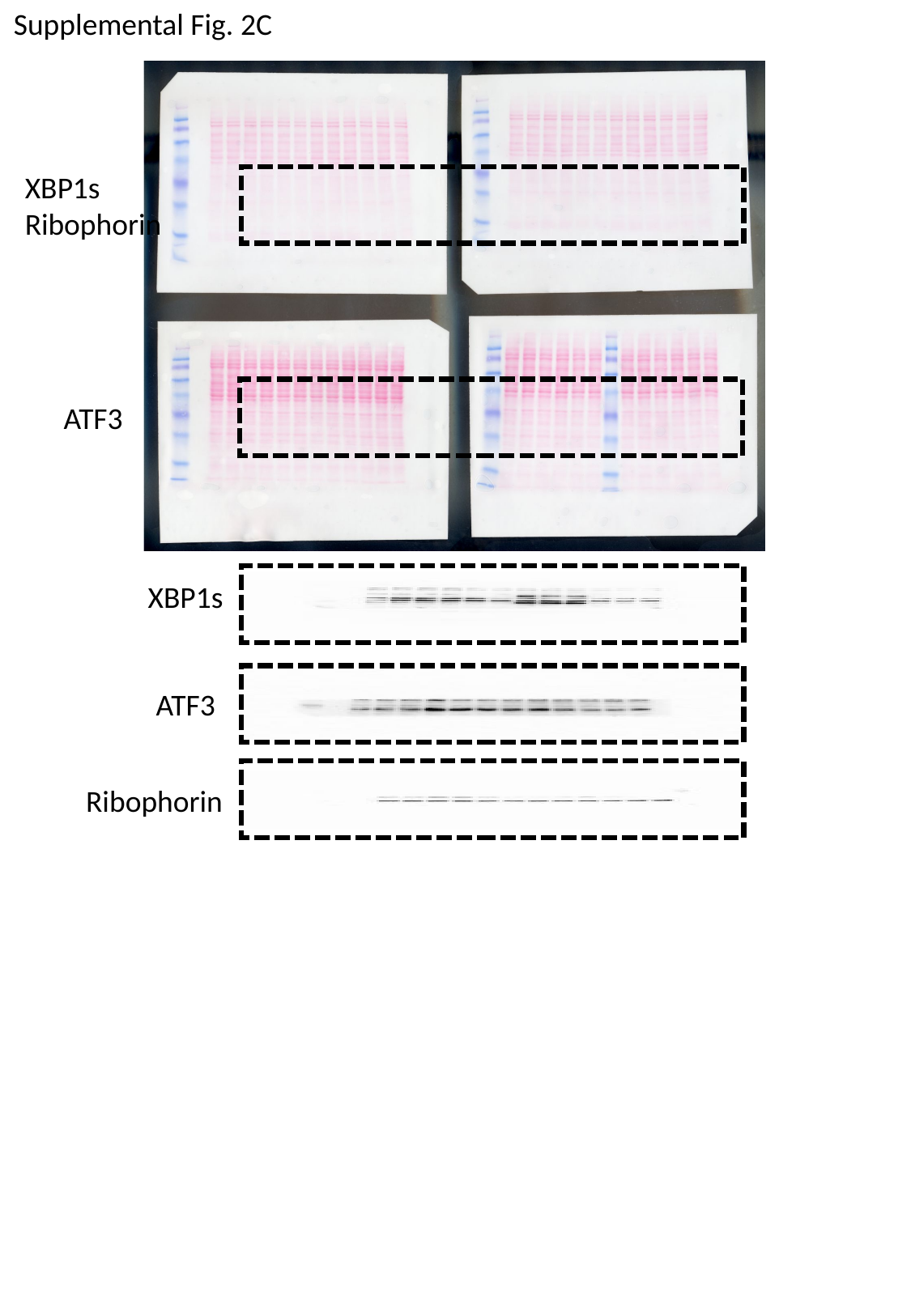

Supplemental Fig. 2C
XBP1s
Ribophorin
ATF3
XBP1s
ATF3
Ribophorin
