# Supplementary material for: Nanosecond pulsed electric fields induce the integrated stress response via reactive oxygen species-mediated heme-regulated inhibitor (HRI) activation
Source: PLoS One. 2020 Mar 10;15(3):e0229948. doi: 10.1371/journal.pone.0229948 (PMC7064201; doi:10.1371/journal.pone.0229948)
Supplement: Attachment — Submitted filename: The manuscript by Hamada et al described the study of nanosecond pulsed electric fields.docx [file pone.0229948.s004.docx]

The manuscript by Hamada et al described the study of nanosecond pulsed electric fields (nsPEFs) on integrated stress response (ISR). They concluded that HRI is the eIF2alpha kinase responsible for the ISR activation by nsPEF-mediated oxidative stress. However, the scope of manuscript is very limited and preliminary. The second arem of ATF4 induction was not investigated in this manuscript. Their study did not increase our knowledge on the mechanisms as how and why nsPEFs may be useful in clinical practice. There is also a concern on the sole approach of using overexpression of single eIF2a kinase. Studies using single KO of each kinase shall be included, if indeed HRI is the sole eIF2a kinase responsible. It is important to note that overexpression of single eIF2a kinase may not be physiological due to the high level of kinase achieved.

The specific comments are:

1. Citations on the literature of HRI are inadequate; missing several important papers on the activation of HRI by oxidative stress and protection of cells against oxidative insults.
2. Fig. 1C, Fig 4A and B

Why is eIF2aP in 4KO+HRI so much lower than WT?

It seems that GCN2 also contributes to increased eIF2aP upon nsPEFs.

3. Fig. 2A, Similarly to Fig. 1C, why is Why is eIF2aP in 4KO+HRI so much lower than WT upon H_2_O_2_ treatment.
4. Fig. 4C, The FACS plots for ROS measurement shall be presented
