# Supplementary material for: Nanosecond pulsed electric fields induce the integrated stress response via reactive oxygen species-mediated heme-regulated inhibitor (HRI) activation
Source: PLoS One. 2020 Mar 10;15(3):e0229948. doi: 10.1371/journal.pone.0229948 (PMC7064201; doi:10.1371/journal.pone.0229948)
Supplement: Attachment — Submitted filename: 3. Response to Reviewers (Hamada et al).docx [file pone.0229948.s006.docx]

Thank you for your letter dated February 10, 2020 inviting us to submit a revised manuscript. We are grateful to the Editor and the reviewers for their critical comments and useful suggestions that have helped us to improve our paper. As indicated in the point-by-point responses below, we amended the figure and the related description in the revised version of our manuscript. As we revised the manuscript with “Track changes” option in WORD, when you turn on “Track Changes”, every change we made to the manuscript will appear as a colored markup.

We hope that the revised version of our paper is now suitable for publication in PLoS One and we look forward to hearing from you at your earliest convenience.

**Reviewer comments:**

*Reviewer #1: The revised manuscript by Hamada et al has addressed all the concerns from the previous review. In particular, the single KO cell lines of HRI and GCN2 were generated and studied. In Furthermore, Activation of HRI in vivo in the Wt Hap1 cells was also demonstrated by phos-tag immunoblot analysis. One minor comment is that T-HRI band in Fig. 2E and F shall be unphophorylated HRI not total HRI. So, it shall be changed to HRI.*

We agreed with the reviewer 1. The phosphorylation states of HRI on Phos-tag SDS-PAGE are now indicated on the right side of the blot with arrowheads. We thank the reviewer for bringing it to our attention.
